# A Multimodal Imaging Study in a Case of Bilateral Thalamic Damage With Multidomain Cognitive Impairment

**DOI:** 10.3389/fneur.2019.01048

**Published:** 2019-10-14

**Authors:** Mariachiara Longarzo, Carlo Cavaliere, Mario Orsini, Liberatore Tramontano, Marco Aiello, Marco Salvatore, Dario Grossi

**Affiliations:** ^1^IRCCS SDN, Naples, Italy; ^2^Department of Psychology, University of Campania Luigi Vanvitelli, Caserta, Italy

**Keywords:** thalamus, stroke, PET, MRI, dementia, connectivity

## Abstract

Severe thalamic injury can determine a particular type of vascular dementia affecting multiple network dysfunctions, considered the central role of thalamus as a hub for afferent and efferent stimuli. A 67-year-old male patient with bilateral thalamic stroke was studied with positron emission tomography, magnetic resonance imaging, and cognitive assessment, performed at baseline and at two follow-up evaluations. A pattern primarily involving thalamo-frontal connections was observed by both PET and tractography analyses. All significant differences between the patient and controls involved the anterior thalamic radiation, one of the major fiber tracts in the fronto-thalamic circuitry. In particular, altered tractography indices of higher radial diffusivity and apparent diffusion coefficient and reduced fractional anisotropy values for the anterior thalamic radiation were reported. In accordance with imaging findings, neuropsychological evaluation demonstrated a multidomain impairment including memory, executive functions, and attention. Additionally, the patients displayed behavioral symptoms, in absence of mood alterations. Multimodal imaging assessment, revealing the metabolic and microstructural alterations that attend to multidomain neuropsychological impairment, demonstrated multiple levels of adaptations to bilateral vascular thalamic injury.

## Introduction

The thalamus is the core diencephalic brain structure subdivided into several nuclei having wide bidirectional connections with cortical and subcortical regions, e.g., cingulate cortex, hippocampus, and amygdala (1). Each portion of the thalamus has specialized connections and functions. Its anterior part receives projections from the mammillo-thalamic tract and is mainly linked with orbitofrontal cortex and cingulate cortex; it is principally involved in memory and emotional disorders. The paramedian nuclei connect with amygdala, prefrontal cortex, globus pallidus, and motor and premotor cortex; its stroke causes reduced consciousness, disinhibition, apathy, and amnesia. The inferolateral territory is responsible for executive functions whereas the posterior portion has projections to occipito-parietal, prefrontal, cingulate, and parahippocampal cortices; no particular behavioral alterations are reported if specifically injured, whereas some cognitive deficits have been described (2).

Literature describes several cases of patients presenting thalamus lesion often affected by cognitive, emotional, and motor deficits; aphasia, agnosia, amnesia, and neglect also occur after thalamic stroke (3–5).

Stenset et al. (6) described a 67-year-old patient with left thalamic lesion who suffered of memory dysfunction; the ^18^fluorodeoxiglucose positron emission tomography (PET) evidenced reduced metabolism in the left anterior thalamus and in frontal, parietal, and temporal lobes in the left hemisphere. Magnetic resonance imaging (MRI) also showed a lesion in the left anteromedian portion of the thalamus.

Differently, Shim et al. (4) used single photon emission computerized tomography (SPECT) and MRI for studying four patients with focal left thalamus stroke, who reported executive dysfunctions in addition to memory impairment. They also found decreased regional cerebral blood flow in frontal, parietal, and temporal gyri and MRI revealed disruption of fibers in the infarcted thalamic area.

Later, SPECT used on 18 patients with left thalamic lesion showed that reduced perfusion observed in both the hemispheres correlated with deficit in executive functions and depression state (5).

More recently, Song et al. (7) described a case report of a thalamic infarction due to artery of Percheron occlusion who presented speech and behavioral alterations.

As shown, available studies in literature used various approach for studying thalamic lesions. It seems that there does not exist a general consensus about methods to perform; therefore, the results are also heterogeneous. Overall, in all cases, thalamic infarct was accompanied by diffuse brain metabolism alterations, and cognitive complaints differed according to lesion site and extension.

In the present study, we examined one patient presenting with bilateral thalamic lesions that we evaluated three times: at baseline (T0) and after 6 months (T1) and 18 months (T2). During the same session, brain PET-CT and MRI were performed with an advanced imaging protocol that includes diffusion tractography (DT).

In addition, neuropsychological assessment to investigate cognitive dysfunctions complained by the patient was performed.

## Materials and Methods

### Case Description

A 67-year-old male patient was admitted to undergo a brain PET-CT and MR for investigating amnestic symptoms he manifested. He referred focal brain hemorrhage in the left superior temporal gyrus in 2016; he complained about memory dysfunctions and visual hallucinations; he also had a persistent left leg pain that makes walking difficult. He was fully cooperative and denied his cognitive problems.

The first PET-CT and MR results (T0) revealed thalamus lesions; therefore, we decided to evaluate the patient, with the same imaging protocol, in two follow-up times, after 6 months (T1) and after 18 months (T2) from injury. Additional MRI exam with contrast agent injection was performed to exclude tumor pathology at baseline. Furthermore, 13 healthy volunteers matched for gender, age, Fazekas score [in regions different from the thalami; (8)], and white matter lesion load (9) (13 males; mean age: 69.31 ± 3.12; total volume lesion: 2.17 ± 1.64), performing the same MR protocol, were selected as the control group.

### Clinical and Neuropsychological Assessment

The patient performed cognitive tests and clinical scales at the baseline evaluation and at the second follow-up time. The global cognitive status was assessed with the Mini Mental State Examination [MMSE; (10)], the Montreal Cognitive Assessment [MoCA; (11)], and the Frontal Assessment Battery [FAB; (12)]. The Clinical Dementia Rating Scale [CDR; (13)] was used to evaluate the degree of dementia severity; it ranges from no dementia (0) to severe dementia (3). Memory is considered the major domain from which they depend subsequent cognitive domain scores depend on orientation, judgment and problem solving, community affairs, home and hobbies, and personal care. The patient also performed Raven's colored progressive matrices (14), phonological and semantic verbal fluency (15, 16), Stroop test (17), Milan constructional apraxia (18), and trail making test (19) in order to collect a comprehensive cognitive profile.

We investigated neuropsychiatric symptoms as depression with the Beck Depression Inventory-II (20), behavioral disorders with the Frontal Behavior Inventory (21), apathy with the Apathy Evaluation Scale (22), and interoceptive consciousness with the Self-Awareness Questionnaire [SAQ; (23)].

### Positron Emission Tomography–Computerized Tomography

Data were acquired using a Discovery 710 PET-CT scanner (GE Healthcare), according to European guidelines (24). The subject was intravenously (i.v.) injected with 250 MBq of [18F]-FDG dose, after a resting period (15 min) in a quiet and dark room. Following the radiotracer injection uptake period of 20–25 min, during which the patient rested with eyes closed, PET data were acquired in sinogram mode for 10 min; matrix size was 256 × 256. PET emission data were reconstructed with ordered subset-expectation maximization (OSEM) algorithm (21 subsets, 4 iterations) and post-filtered with a three-dimensional isotropic gaussian of 4 mm at FWHM. Attenuation correction was performed using CT-based attenuation maps derived from a CT scan (140 kV, 300 mA, 3.75 mm thickness).

### Magnetic Resonance Imaging

In the same day, a 3-T Biograph mMR tomograph (Siemens Healthcare, Erlangen, Germany) was used, equipped with a 12-channel head coil, after 1 h from radiotracer administration. The MRI protocol included morphological volumetric T1 (TR: 2,400, TE: 2.25, voxel: 0.8 mm^3^ isotropic, matrix: 256 × 256), T2 (TR: 3,370, TE: 563, voxel: 0.8 mm^3^ isotropic, matrix: 256 × 256), Fluid Attenuated Inversion Recovery (TR: 5,000, TE: 354, voxel: 1 mm^3^ isotropic, matrix: 192 × 192) as well as techniques like DTI (TR: 3,851, TE: 84.2 voxel: 2 mm^3^ isotropic; 71 directions; *b* value max: 1,500, matrix: 128 × 128). Foam wedges were used to minimize movement artifacts, and the patient held eyes closed during the resting-state scan.

### Image Analysis

#### PET-CT

PET images were processed with Scenium tool, of Syngo.Via software (Siemens Healthcare, Erlangen, Germany). It normalized and parceled with a standardized PET database of healthy subject matched for age [Database: FDG-PET Biograph HD, age 46–79, Cerebellum-Vermis; Atlas: Automated Anatomical Labeling; (25)].

#### DT

White matter lesions were segmented by the lesion prediction algorithm (9) as implemented in the LST toolbox version 2.0.15 for Statistical Parametric Mapping 12 (SPM12).

The diffusion tensor images were elaborated with software FSL and MrTrix by the following processing steps: denoizing (26), motion and eddy current correction (27), DWI reslicing to the T1 space by trilinear interpolation (28), ACT (anatomical–constrained tractography) (29), and deterministic tractography reconstruction (30).

Anterior thalamic radiation segmentation, between thalamus and homolateral frontal cortex, was performed by TrackVis (Version 0.6.1) on control group and on each patient's time points. A volume of interest (VOI) on thalami and a region of interest (ROI) on frontal region were applied to reconstruct anterior thalamic radiation. Thalamus VOIs were defined by considering the segmentation of subcortical areas resulting from standard FreeSurfer (v.5.1) pipeline (31) on volumetric T1. Frontal region ROIs were defined as the plane passing perpendicularly the frontal lobe and tangent, on sagittal plane, to genus corpus callosum. Consequently, the fractional anisotropy (FA), apparent diffusion coefficient (ADC), axial diffusivity (AD), and radial diffusivity (RD) values were calculated. A Bayesian comparison was performed for all values between each patient's time points and the control group with *singlebayes* tool (32, 33).

Finally, a voxel-wise two-sample *t*-test was performed on FA, AD, ADC, and RD maps with SPM12 software (https://www.fil.ion.ucl.ac.uk/spm/) between each patient's time points and the control group. Briefly, maps were normalized using the high-resolution FMRIB58_template in the Montreal Neurological Institute (MNI) space applied to consider only the white matter, including only the FA values > 0.21 (34).

A threshold of *p* < 0.05 with false discovery rate (FDR) as multiple comparison correction was considered as significant.

## Results

### Clinical and Neuropsychological Assessment

Neuropsychological evaluation evidenced a multidomain impairment. Global cognitive status was compromised, as evidenced by performance on MMSE, FAB, and MoCA. Results of CDR suggest the presence of a mild degree of dementia. The patient failed in tests for executive functions, and attention performance was borderline with respect to cutoff. Visuo-spatial abilities and fluid intelligence were preserved from deterioration. No depressive and apathetic symptoms resulted from the questionnaires. Interoceptive consciousness was inferior to normative data; finally, the patient presented several behavioral dysregulation symptoms.

Follow-up neuropsychological assessment confirmed cognitive dysfunction, but no deterioration was observed. Executive functions are the cognitive domain mainly impaired in our patient. No depressive or apathetic symptoms occurred. Interoceptive consciousness was significantly inferior with respect to normative data; behavioral alterations diminished over time with respect to first clinical evaluation. Cognitive and clinical tests are summarized in Table 1.

**Table 1:** Summary of neuropsychological and clinical results at the baseline and follow-up assessments.

	**T0 raw score**	**T2 raw score**	**Cut-off**	**Cognitive domain**
MMSE	20.2	21.2	23	Global cognitive status
FAB	10	12	13.5	Executive functions screening
MoCA	19	18	26	Mild cognitive impairment
CDR	1	1	–	Dementia
Ravens' progressive colored matrices ‘47	26	26	18.9	Fluid intelligence
Phonological fluency	10	22	17.3	Executive functions
Semantic fluency	14.5	16.5	33.2	
Stroop test	1: 17” (t)	1: 20”		
	2: 19” (t)	2: 22”		
	3: 48” (t)	3: 50”	T:36.92	
	7 errors	7 errors	E:4.24	
Milan constructional apraxia	12	11	8	Visuo-spatial abilities
TMT	A:77	A:87	A <94	Attention
	B:249	B:132	B <283	
	B-A:172	B-A:45	B-A <186	
BDI-II	7	7	14	Depression symptoms
FBI	26	9	20	Behavioral symptoms
SAQ	22	16	27	Interoceptive consciousness
APATHY EVALUATION SCALE	33	34	38	Apathy

*MMSE, Mini Mental State Examination; FAB, Frontal Assessment Battery; MoCA, Montreal Cognitive Assessment; CDR, Cognitive Dementia Rating; TMT, Trail Making Test; BDI-II, Beck Depression Inventory-II; FBI, Frontal Behavior Inventory; SAQ, Self-Awareness Questionnaire*.

## PET-CT

**Figure 1:**
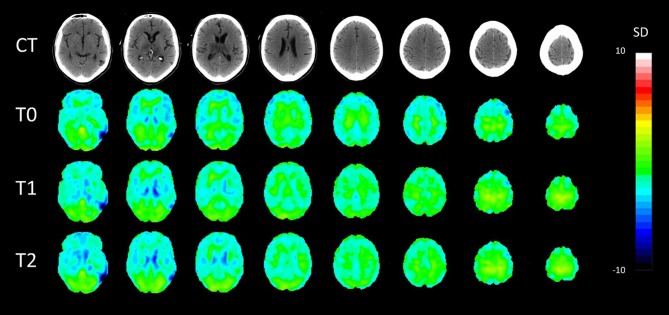
Axial patient normalization map PET with co-registered CT in the three time points. SD, standard deviation colored scale. Slices were taken with 8 mm distance.

PET-CT analyses confirmed bilateral thalamic hypometabolism, more pronounced on the left side. In the left thalamus, hypometabolism was increased between baseline and first follow-up and stationary between the two follow up (T0, *Z* = −4.4; T1, *Z* = −5.6; T2, *Z* = −5.5). In the right thalamus, there was a significant hypometabolism only in the second time point (T2, *Z* = −4.3). Moreover, in the left inferior temporal gyrus, there was hypometabolism at the baseline and second follow-up (T0, *Z* = −4.1, T2, *Z* = −4.1) but not in the first (T1, *Z* = −3.3). In the gyrus rectus, the decreased metabolism was present in the second and third acquisitions (T1, *Z* = −4.3, T2, *Z* = −4.3). Pet-ct results are presented in Figure 1. The other brain regions with a significant *Z* score are summarized in Table 2.

**Table 2:** Significant metabolic brain areas resulting from PET-CT between time-points patient and database control.

	**Z score**		
**Brain area**	**T0**	**T1**	**T2**
Heschl gyrus (L)	−3.5	−3.6	−4.1
Inferior temporal gyrus (L)	−4.1	−3.3	−4.1
Middle temporal gyrus (L)	−3.8	−3.3	−4.1
Mesial temporal lobe (R)	−1.7	−2.6	−4.2
Superior frontal gyrus medial orbital (L)	−3.0	−3.0	−4.2
Superior frontal gyrus orbital part (R)	−3.7	−3.2	−4.2
Basal ganglia (L)	−3.5	−3.5	−4.3
Corpus striatum (L)	−3.5	−3.4	−4.3
Parahippocampal gyrus (L)	−1.9	−2.6	−4.3
Thalamus (R)	−3.0	−4.3	−4.3
Mesial temporal lobe (L)	−1.8	−2.5	−4.4
Gyrus rectus (L)	−3.9	−4.0	−4.5
Olfactory cortex (R)	−2.9	−3.2	−4.5
Gyrus rectus (R)	−3.7	−3.8	−4.7
Superior frontal gyrus medial orbital (R)	−3.4	−3.3	−4.7
Caudate nucleus (L)	−3.7	−3.7	−5.1
Thalamus (L)	−4.1	−5.6	−5.5

## DT

**Figure 2:**
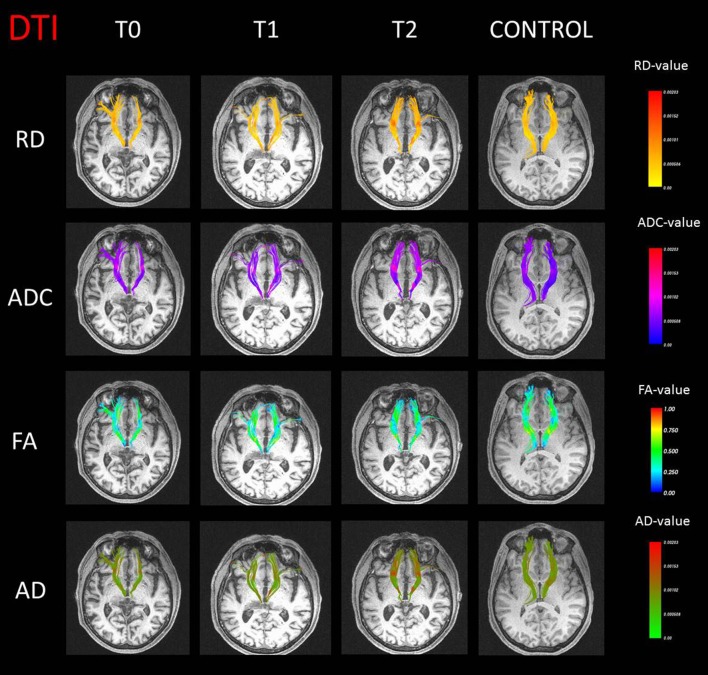
Anterior thalamic radiation streamline representation with values of radial diffusivity (RD), apparent diffusion coefficient (ADC), fractional anisotropy (FA), and axial diffusivity (AD) in false color maps for the case (three different time points) and a representative control subject.

Significant differences were found between patient and controls for the diffusion tractography indices. A difference on RD for the right anterior thalamic radiation in T0 (*p* = 0.03) was found when compared to the control group. The ADC (T0: *p* = 0.007; T1: *p* = 0.006) and RD (T0: *p* = 0.01; T1: *p* = 0.02) values were significantly increased in the left anterior thalamic radiation. Furthermore, at T1, an increase on ADC (*p* = 0.006) and RD (*p* = 0.001) and a decrease on FA (*p* = 0.02) were observed compared to control subjects, whereas no significant results were obtained on AD index. All tractography maps are presented in Figure 2.

Finally, voxel-wise analyses between normalized DT maps of control subjects and the T2 did not show significant differences.

## Discussion

In this longitudinal study, we evaluated three times, in about 2 years, a patient who presented with vascular dementia marked by severe bilateral thalamic lesion and that performed PET/CT and MRI. While focal unilateral thalamic infarction more frequently occurs, diffuse bilateral lesion, as the case presented in this work, is more uncommon and can determine a particular type of vascular dementia that, involving a strategic area for many neural networks (35), determines dysfunction of entire networks, not simply areas.

PET analysis highlighted a hypometabolic pattern that involved the thalami and several cortical areas; thalamic hypometabolism was quite diffuse, not permitting to identify a specific component more impaired with respect to others. Associated areas afferent primarily to temporal and frontal lobes, and representing projection areas of the thalami, showed significant reduced metabolism, as in the case of the left parahippocampal gyrus.

Since the medial dorsal and anterior nuclei of the thalamus are related to the hippocampus, they are considered to play a role in formation of new memories and project to afferent pathways from the temporal to the frontal lobe (36). This reduction might explain memory disturbances presented by the patient and resulting from the neuropsychological assessment. In this regard, the CDR revealed difficulties in detaining and recalling information regarding the recent past instead of well consolidated remote information. Such deficits we observed, attributable to an onset of dementia, are in line with von Cramon et al. (37) and Schmahmann (36) who reported that thalamus lesions can impair recent memory. Also, Serra et al. (38) observed memory impairment in two patients with bilateral thalamic lesions they studied by structural MRI; the first subject showed a mainly right damage and complained about memory deficits; the other one had a more symmetrical lesion and showed executive dysfunctions in addition to memory impairment.

Hypometabolism in the right thalamus seems to be a marker of subcortical vascular mild cognitive impairment, whereas the patients with amnestic mild cognitive impairment show hypometabolism mainly in left parahippocampal gyrus and orbitofrontal regions (39). Moreover, the ischemic interruption of frontal subcortical circuits also affects mood and behavior and contributes to the cognitive impairment of subcortical vascular dementia (40, 41). Our patient presents a mixed condition, where the frontal lobe is involved due to the bilateral superior frontal gyrus reduced uptake compared to control subjects. The superior frontal gyrus has anatomical connections with the thalamus (42), its posterior part is generally mainly activated by motor tasks, whereas the lateral part is involved in working memory and attention and the medial part, afferent to the default-mode network, is deactivated by cognitive-related processing (43). Frontal involvement could explain behavioral symptoms presented by the patient in terms of lack of flexibility, disorganization about complex activities, perseveration, and irritability. Reduced metabolism also involved the temporal lobe, in particular temporal and Heschl gyri. Middle and inferior temporal gyri subserve language and semantic memory processing, visual perception, and multimodal sensory integration. Temporal hypometabolism is in accordance with impaired cognitive performances, especially on executive functions, in particular semantic fluency, since phonemic and semantic fluency are usually used to test neurologic damage but semantic component is more impaired following temporal lesions. Therefore, medial temporal hypometabolism found here points for a dementia diagnosis, since the patient showed a widespread impairment, revealing multidomain cognitive problems. He had altered global cognitive status and executive functions, in both the baseline and follow-up evaluation, even if the patient performed both performances slightly better on the follow-up time. The MoCA test results were in accordance with MMSE and FAB performances, confirming cognitive impairment. Among the batteries, the CDR, which is specific for characterizing level of memory decline, showed the presence of a mild level of dementia. More specifically, single tests that explore single cognitive domains showed that executive functions appeared partially altered, whereas attention, apraxia, and fluid intelligence were preserved from deterioration. On clinical tests, the patients displayed no depressive or apathetic symptoms and a normal level of interoceptive consciousness.

Levasseur et al. (44), among the first, studied seven cases of patients with bilateral thalamic infarcts by PET, showing diffuse cortical hypometabolism and associated amnesia. De Falco et al. (45) reported a patient with severe memory loss and apathy. MRI showed bilateral thalamic damage of posterior and medial areas, involving part of the pulvinar, more evident for the right thalamus. Six months later, a severe memory impairment was still evident and both MRI and SPECT findings were unchanged.

Sandson et al. (3) described the case of a patient who presented with left medial thalamic infarction evident from computerized tomography, electroencephalography, MRI, and SPECT that evidenced frontal lesions and presented with several personality changes and cognitive impairments mainly in memory, language, and executive functions domains. The authors hypothesized that the deficit of complex behavioral functions resulted from injury of the dorsomedial nucleus of the thalamus involved in the frontal network subserving these abilities.

Additionally, reduced glucose metabolism in basal ganglia, striatum, and caudate nucleus was detected. Basal ganglia are the core structures of extrapyramidal motor system, but also are involved in pathways subtending to emotional, motivational, and cognitive functions. The striatum receives inputs from cortical areas and, via the thalamus, projects to prefrontal, premotor, and supplementary motor areas. The circuit involving basal ganglia, thalamus, and cortical areas maintains movement organization, mainly involuntary and stereotyped (1). The patient complained about some difficulties in walking and pain to the right leg; therefore, we hypothesize that abnormal uptake in these areas, related to vascular outcomes he had, could explain the motor symptoms he suffers.

In line with metabolic results, analyses of tractography evidenced altered connections between the thalamus and frontal regions. All significant differences between the patient and controls involved the anterior thalamic radiation, one of the major fiber tracts in the fronto-thalamic circuitry that connects the prefrontal lobe to the anterior and dorsomedial thalamic nuclei. Reduced anisotropy and augmented diffusivity in the anterior thalamic radiation were detected in the patient, signaling a structural change of white matter (46), both in baseline and in the first follow-up evaluation. Furthermore, higher RD and ADC and reduced FA values for the anterior thalamic radiation were in line with the evidence of thalamic lesion.

Almost all imaging results presented on patients with thalamic lesions focus on brain metabolism, largely ignoring structural impairments evident with the diffusion tractography. In the present work, structural changes have been observed in the diffusion tractography index, in absence of results from voxel-wise analysis. The lack of significant differences in the latter analysis could be explained by the fact that voxel-based analysis and fiber tractography are methods using different approaches. It is plausible that results from the voxel-wise analysis were affected by an ineffective registration between subjects, since it is a fundamental prerequisite for voxel-wise group comparison. Conversely, in diffusion tractography, the tracts can be delineated without relying on subject registration. However, specific a priori regions of interest or specific tracts need to be selected for comparison, as we have done.

The most striking finding of the present work is the association between structural and metabolic changes within the fronto-thalamic circuitry in our patient. Multimodal imaging assessment longitudinally demonstrated adaptations to bilateral vascular thalamic injury at multiple levels, revealing the metabolic, functional, and microstructural alterations attending to multidomain neuropsychological impairment.

## Data Availability Statement

All datasets generated for this study are included in the manuscript.

## Ethics Statement

The studies involving human participants were reviewed and approved by IRCCS Pascale Ethical Committee. The patients/participants provided their written informed consent to participate in this study. Written informed consent was obtained from the individual(s) for the publication of any potentially identifiable images or data included in this article.

## Author Contributions

CC interpreted the results and wrote the paper together with ML who performed neuropsychological data collection, analyses, and interpretation. MO performed MRI acquistion and tractography and PET analyses. LT performed neuroimaging acquisition and analyses of the new sample of control subjects. DG developed the study concept and design and made manuscript revision. MA and MS revised the manuscript and approved the draft.

### Conflict of Interest

The authors declare that the research was conducted in the absence of any commercial or financial relationships that could be construed as a potential conflict of interest.
